# Genetic consequences of social structure in the golden-crowned sifaka

**DOI:** 10.1038/s41437-020-0345-5

**Published:** 2020-08-13

**Authors:** Bárbara Parreira, Erwan Quéméré, Cécile Vanpé, Inês Carvalho, Lounès Chikhi

**Affiliations:** 1 Instituto Gulbenkian de Ciência, Rua da Quinta Grande, 6, 2780-156 Oeiras, Portugal; 2 Laboratoire Évolution & Diversité Biologique (EDB UMR 5174), CNRS, IRD, UPS, Université de Toulouse Midi-Pyrénées, Toulouse, France; 3 Present Address: ESE, Ecology and Ecosystems Health, Ouest, INRAE, Rennes, France; 4 Present Address: Equipe Ours, Unité Prédateurs et Animaux Déprédateurs et Exotiques, Office Français de la Biodiversité, Impasse de la Chapelle, 31800 Villeneuve de Rivière, France

**Keywords:** Social evolution, Evolutionary biology

## Abstract

Many species are structured in social groups (SGs) where individuals exhibit complex mating strategies. Yet, most population genetic studies ignore SGs either treating them as small random-mating units or focusing on a higher hierarchical level (the population). Empirical studies acknowledging SGs have found an overall excess of heterozygotes within SGs and usually invoke inbreeding avoidance strategies to explain this finding. However, there is a lack of null models against which ecological theories can be tested and inbreeding avoidance quantified. Here, we investigate *inbreeding* (deviation from random mating) in an endangered forest-dwelling pair-living lemur species (*Propithecus tattersalli*). In particular, we measure the inbreeding coefficient (*F*_IS_) in empirical data at different scales: SGs, sampling sites and forest patches. We observe high excess of heterozygotes within SGs. The magnitude of this excess is highly dependent on the sampling scheme: while offspring are characterised by a high excess of heterozygotes (*F*_IS_ < 0), the reproductive pair does not show dramatic departures from Hardy–Weinberg expectations. Moreover, the heterozygosity excess disappears at larger geographic scales (sites and forests). We use a modelling framework that incorporates details of the sifaka mating system but does not include active inbreeding avoidance mechanisms. The simulated data show that, although apparent “random mating” or even *inbreeding* may occur at the “population” level, outbreeding is maintained within SGs. Altogether our results suggest that social structure leads to high levels of outbreeding without the need for active inbreeding avoidance mechanisms. Thus, demonstrating and measuring the existence of active inbreeding avoidance mechanisms may be more difficult than usually assumed.

## Introduction

In many vertebrate species, individuals live in social groups (SGs) with variable kinship structure, complex mating strategies, and sex-biased dispersal (Clutton-Brock 2016). The large variety of species that live in groups include many non-humans primates, rodents, bats, cetaceans, fish and birds (Clutton-Brock 2016; Rubenstein and Abbot 2017). Within these, social organisations can range from temporary pair-bounded aggregations to stable complex societies, exhibiting cooperative breeding or complex dominance hierarchies for example (Clutton-Brock 2016). Sociality has been mostly interpreted as an adaptive mechanism (Silk 2007; Clutton-Brock 2009, 2016) within the context of the kin selection theoretical paradigm. According to this paradigm, individuals may gain indirect (inclusive) fitness benefits by enhancing the fitness of others through helping and cooperating with relatives (that carry copies of the same genes; Hamilton 1964). Several studies propose that, at low population inbreeding loads, inclusive fitness benefits should induce individuals to prefer mating with relatives (Lehmann and Perrin 2003; Kokko and Ots 2006). Indeed, in some species animals do prefer to mate with kin rather than with unrelated individuals (Rioux-Paquette et al. 2010; Olson et al. 2012; Szulkin et al. 2013; Jacob et al. 2016). From a population genetics perspective, the consequence of mating among related individuals is increased homozygosity, which can expose recessive deleterious alleles causing inbreeding depression (Charlesworth and Charlesworth 1999). Inbreeding avoidance is thus seen as an important evolutionary mechanism (Szulkin et al. 2013). Sex-biased dispersal and differences in dispersal distances between sexes are often invoked as inbreeding avoidance mechanisms (Greenwood 1980; Lawson Handley and Perrin 2007). However, this usually relies on verbal arguments that make a link between behavioural observations and outbreeding without quantifying kin recognition and dispersal as means of inbreeding avoidance (see Perrin and Mazalov 1999; Roze and Rousset 2005 for theoretical discussions on inbreeding avoidance as the reason for dispersal). Although inbreeding avoidance and inbreeding preference are typically envisioned in a selective context (Silk 2007; Lukas and Clutton-Brock 2011; Clutton-Brock 2016), there is an increasing number of studies challenging this view, highlighting the limitations of current inbreeding theories and models and thus calling for new neutral models (Szulkin et al. 2013; Parreira and Chikhi 2015).

Inbreeding is often estimated through Wright’s inbreeding coefficient (*F*_IS_), a parameter that was developed under the classical population genetics framework (Wright 1951). In practice, *F-*statistics are a tool for describing the partitioning of genetic diversity within and among (sub) populations. *F*_IS_ measures the deviation from Hardy–Weinberg proportions within demes. Negative values indicate an excess of heterozygotes while positive values indicate a deficit. Under this framework, the latter means that mating occurs more often between more closely related individuals than between individuals drawn at random from the (sub)population. *F*_ST_ measures the genetic variance in allelic frequencies among demes, describing the mean reduction in heterozygosity of a deme relative to the total due to genetic drift. This framework ignores social structure and envisions structured populations as a network of panmitic demes, which are considered the smallest level of population structure. When species are socially structured, the smallest unit of population structure is the SG. SGs typically comprise individuals of several overlapping generations, with complex mating strategies and often sex-biased dispersal. Social structure was addressed in a theoretical mathematical framework by Chesser (1991a, b) and Chesser et al. (1993), who extended Wright’s *F*–statistics to describe the apportionment of genetic diversity in populations subdivided in SGs. Under this framework, *F-*statistics are derived as a function of co-ancestries of genes in individuals within and among SGs. Chesser (1991a, b) and Chesser et al. (1993) have shown that *F*_IS_ is expected to be negative within SGs. This means that we expect to observe a negative *F*_IS_ within SGs even in the absence of inbreeding avoidance mechanisms, either those that are active (such as kin recognition and extra-group copulations) or those interpreted as “passive inbreeding avoidance” (e.g. sex-biased dispersal; Chesser 1991b; Sugg and Chesser 1994; Sugg et al. 1996; Di Fiore 2012; Parreira and Chikhi 2015). The fact that negative *F*_IS_ values are expected within SGs also shows that, despite the fact that individuals within SGs are highly related (through co-ancestry maintained by the pedigree), a high relatedness may not be translated into a loss of genetic diversity (inbreeding measured by drift as change in heterozygosity). This is highly counterintuitive because under the classical population genetics framework small units (demes) are expected to lose genetic diversity by drift and accumulate *inbreeding* (co-ancestry by drift; see Wakeley and Wilson 2016 for a review on the several concepts of identity-by-descent).

We have proposed a new framework that explicitly models the subdivision of species into SGs, incorporates mating systems, and can be used as a null-model to test inbreeding avoidance strategies (Parreira and Chikhi 2015). Under this framework, populations are modelled as a network of SGs, which are age-structured units where individuals mate according to different strategies (e.g. monogamy, polygyny) without any mating bias against kin (Parreira and Chikhi 2015). Our previous findings based on this framework were purely theoretical and based on an *n*-island type of structure that incorporates structure but ignores space (i.e., every SG is connected to all others in the overall population and thus all SGs are at the same “distance” from each other). Previous results were based on simulated scenarios and no real species ecological and genetic data were used.

The aim of the present study is to understand the role of the social structure in the genetic diversity of an endangered primate species, the golden-crowned sifaka *(Propithecus tattersalli*, Simons 1988). Previous genetic studies have focused on the patterns of genetic differentiation across the landscape or on the demographic history of the species (Quéméré et al. 2010a, b, 2012). Quéméré et al. (2010a) reported a bias towards negative *F*_IS_ values (ranging from −0.01 to −0.18) at the level of forest fragments. However, the origin of this pattern has not been investigated. Here, we ask whether outbreeding is a consequence of population structure in the form of SGs and dispersal. In this study we apply our previously developed modelling framework to the golden-crowned sifaka (Parreira and Chikhi 2015). We use the simulations as a null model to predict *F*_IS_ values for a population subdivided in SGs where mating occurs according to monogamy (*P. tattersalli* mating system) and without inbreeding avoidance by mate choice through kin recognition. As such, if sifakas have developed mechanisms to avoid inbreeding, we expect *F*_IS_ values to be more negative in the real population than in the simulated one. Our aim is to understand the effect of social structure on the levels of inbreeding in this species and to provide hints for its biological interpretation.

## Materials and methods

### Study population and sampling strategy

**Fig. 1:**
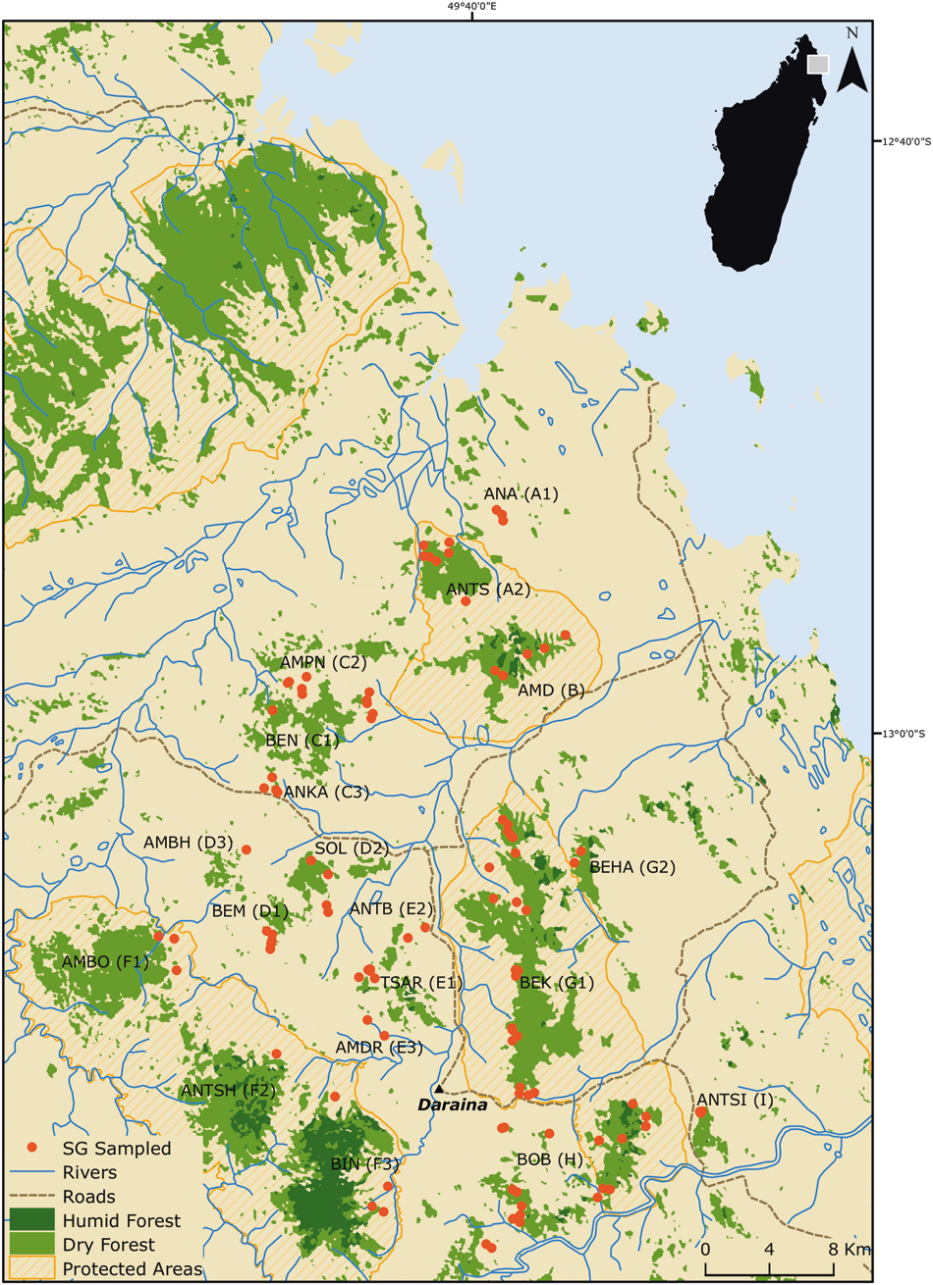
Map of the Loky-Manambato region showing the location of sampling sites. The golden-crowned sifaka inhabits relatively isolated forest patches in a restricted range within the Loky-Manambato region, in northeastern Madagascar. Red dots show the location of sampled SGs. Letters identify forest fragments, and are followed by a number which identifies the sampling site.

The golden-crowned sifaka is a social lemur species from the *Indriidae* family (Simons 1988). It is only found in a restricted and fragmented forest habitat in the Loky-Manambato region of northeastern Madagascar (Quéméré et al. 2010a, b, Fig. 1) and has been classified as critically endangered in IUCN’s last assessments (e.g. Andriaholinirina et al. 2014, Semel et al. 2020). Studies on the ecology and social structure of sifakas are still limited, but there is a long-term study on *Propithecus verreauxi* that describes sifakas as diurnal animals living in SGs that contain from two to ten individuals (Lawler et al. 2003; Lewis 2008). One single male and one single female are thought to monopolise reproduction, although groups may include other (sub)adults which are thought to be pre-dispersal natal individuals (Meyers 1993). Females are more philopatric than males; within groups adult females can relate to each other as mother–daughter or as sisters, while males are thought to leave the natal group once they reach sexual maturity (Meyers 1993).

Faecal material from 224 individuals belonging to 104 SGs was collected during two field missions in 2006 and 2008 at 19 sites in the nine main forest fragments of the species’ distribution range (Quéméré et al. 2010a, b, Fig. 1). These data were already published and are available at the Dryad Digital Repository (doi.org/10.5061/dryad.8f45n). The geographic location of all samples was recorded by GPS and is presented in Fig. 1. The sample collection, faecal DNA extraction and genotyping procedures were performed by Quéméré et al. (2010a, b) and protocols are extensively described therein. In short, faecal extraction was performed using a protocol adapted from Vallet et al. (2008) and all individuals were genotyped for 13 microsatellite loci. Samples were genotyped using a sequential replicate-based approach according to which, in order to ensure the reliability of the genotyping, an allele is recorded only if amplification is unambiguous at least twice for heterozygous genotypes and at least three times for homozygous genotypes (Frantz et al. 2003). Two amplifications are initially performed and, if necessary, further amplifications are sequentially added (up to seven successful PCRs) until a consensus genotype is reached. Genotyping error rates (allele dropout and false error rates) and mean quality index for each locus across individuals (QI) were calculated following Miquel et al. (2006) and are provided in Quéméré et al. (2010b).

The number of sampled individuals within SGs varied from one (25 out of the 104 SGs) to four, but only groups composed of two or more individuals were included in the analysis performed at the SG level (see below). Within the largest forest fragments, SGs were sampled in several sites (Fig. 1). Although the geographic limits of these “sites” are somewhat arbitrary, they consist of an agglomeration of SGs geographically close to each other, and would correspond to how a “sampling location” is usually defined in other empirical population genetics studies.

### Simulation approach

**Fig. 2:**
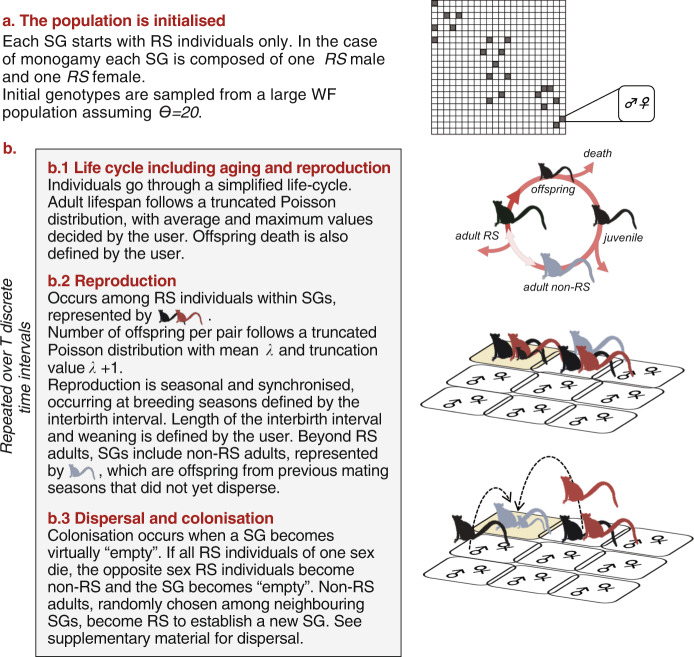
General assumptions underlying the SGs modelling framework. **a** The simulated population consists of a large network of 22 × 22 SGs. Individuals can move to their four neighbouring SGs. Grey squares identify the position of SGs from where individuals were sampled in order to calculate *F-*statistics. **b** Each SG is simulated as being composed of two reproductive status individuals (RS) identified by large figures (red females and black males), offspring and juveniles, identified by small figures, and non-RS adult individuals identified by grey figures. For *Propithecus tattersalli* the number of RS individuals is fixed to one male and one female. Within SGs individuals undergo a simplified life-cycle including aging (b.1) and reproduction (b.2). Individuals colonize and disperse to neighbouring SGs (b.3). This simplified life-cycle is repeated over a pre-defined number of time intervals. See supplementary material for further details.

We used a framework previously developed by us (Parreira and Chikhi 2015, where a detailed explanation of the model can be found). In a few words, individuals are not modelled as members of populations but as members of a network of SGs. SGs are age-structured units wherein diploid dioceous individuals reproduce according to specific mating strategies (e.g. monogamy, polygyny, etc., see below) and among which individuals can disperse (Fig. 2). This framework is an individual-based forward time model where each individual is explicitly characterised by its sex, reproductive status, age and genotype. Individuals undergo a simplified life-cycle involving different age classes: new borns, juveniles, and adults (reproductive status—RS or non-reproductive status—non-RS, see below). This allows for a great flexibility in simulating attributes of primate social systems, such as long living, slow reproduction, strong variance in reproductive success, dominance hierarchies, etc. Under this framework, SGs are units in which generations overlap. This is thus fundamentally different from the classical Wright–Fisher (WF) approach that envisions populations as a network of random-mating units (demes) with non-overlapping generations that exchange migrants according to a pre-defined rate (Wright 1943). Reproduction occurs among individuals living in the same SG according to different strategies such as monogamy, polygyny, etc., without any active kin discrimination behaviour. Only a limited number of adult individuals take part in reproduction and these are identified as RS individuals. RS individuals are a fixed number of males and females within each SG and the ratio RS males to RS females characterises the mating system. In the simulations performed here, we set RS individuals to one in both males and females so that there is only one mating pair per SG — monogamy as in the golden-crowned sifaka. Male–female mating pairs are formed without taking relatedness into account, i.e., no inbreeding or outbreeding.

To account for spatial structure, we simulated a geographical area, represented as a grid of 22 by 22 SGs (total of 484 SGs with bounded edges). This grid is equivalent to a stepping-stone model except that the units are SGs instead of demes (Fig. 2a). In practice, this set of interconnected groups can be seen as a single forest fragment. We considered that individuals could disperse to the closest neighbouring SGs in agreement to what has previously been suggested for this species. Quéméré et al. (2010a) have found significant isolation-by-distance and spatial autocorrelation at a small spatial scale (<1.5 km), which suggested dispersal is most likely to be restricted among neighbouring groups, a finding that is in agreement with behavioural observations for this species (Meyers 1993). In the model, dispersal will only occur if the individuals can become RS and reproduce in a neighbouring SG. That is, we considered actual dispersal events rather than applying a constant dispersal rate. Since RS individuals maintain their status until death, breeding vacancies within a SG only become available due to death events. When all RS individuals from one sex die, RS individuals of the remaining sex lose the reproductive status (becoming non-RS) and the SG virtually vanishes. A new SG will be founded by individuals that move from a neighbouring SG in order to establish themselves as new RS. New RS individuals are chosen at random among non-RS according to the predefined connections in the network. A SG may include adults that were not yet able to disperse (non-RS) and that may eventually not disperse at all during their life if no such opportunity appears. In nature, sifaka males disperse or are evicted from their natal group once they reach adulthood, and thus adult non-breeding males (non-RS individuals) are usually not part of a SG. A flow chart summarizing the main assumptions of the modelling framework is displayed in Fig. 2.

There is scarce information on the life-history traits of the golden-crowned sifaka, but it is known that females usually give birth to a single offspring once every two years (Garbutt 2007) and that individuals can live up to 20 years in captivity (Weigl 2005). Age of sexual maturity is not known with precision but females of a closely related sifaka species (*P. verreauxi*) are known to become sexually mature two or three years after birth (Richard et al. 2006). These ecological data were used as a starting point to define the most likely values for the birth interval, number of offspring, and age of first reproduction in the model. Because values for many demographic parameters reported in literature (lifespan, sexual maturity, weaning age, etc.) are uncertain for the golden-crowned sifaka, we tested 1800 different parameter combinations in a number of exploratory simulations by considering a realistic range of values for each parameter (Table S1). For each parameter combination we produced 100 replicates. We retained the parameter combination that led to a demographically stable population (not causing population crash) and that, at the same time, resulted in group sizes similar to those observed in nature (up to ten individuals, see above). Specifically, we selected a parameter combination that led to two up to 20 individuals per SG, including the reproductive pair, their offspring, juveniles, and non-reproductive adults (which usually do not stay in real sifaka SGs).

In order to measure genetic variation within and among SGs, we randomly sampled 22 social units along the main diagonal of the simulation matrix (Fig. 2a). This sampling design incorporates the full range of possible distances among SGs, allowing us to account for the effect of isolation-by-distance (Wright 1943). We sampled four individuals per SG whenever possible. Individuals were randomly sampled according to different sampling schemes: (1) among juveniles, RS, and non-RS females (hereafter denominated as random sampling), (2) juveniles, (3) RS individuals only (only two individuals were sampled in this case). We chose to ignore non-RS male adults because in nature these individuals disperse as soon as they reach adulthood and are usually not found within SGs. A modelling framework that incorporates ecological data for a specific social species allows obtaining the expected (null) *F*_IS_ distribution for a given species of interest. Differences between empirical and theoretical distributions provide us with information on the amount of inbreeding/outbreeding generated by matings among related/unrelated individuals beyond that generated by the subdivision of populations in SGs.

### Assessment of population subdivision and genetic structure

The genetic diversity and structure were described using the heterozygosities estimated according to Nei (1978) and the *F-*statistics estimators calculated according to Weir and Cockerham (1984). We used two *F-*statistics: *F*_IS_, which measures for deviations from Hardy–Weinberg equilibrium as the correlation of alleles within individuals in relation to that of the sub-population unit considered (SG, “site” and forest fragment); and *F*_ST_, which measures for genetic differentiation. In the empirical study *F-*statistics and heterozygosities were measured using the GENETIX 4.05.2 software (Belkhir et al. 1996). Statistics were measured at three different scales: the SG (*N* = 76, only groups where two or more individuals have been sampled were considered), the sampling site (*N* = 19 locations comprising several neighbouring SGs), and the forest fragment (*N* = 9 areas comprising several sites that are separated by unsuitable habitat; see Fig. 1). Statistics measured at the “site” and fragment scales, as usually done in population genetics studies, ignore the information about the SG of origin of each individual and pool together individuals sampled from the same “site” and fragment. In the simulation study*, F-*statistics were estimated within SGs and at the fragment scale by pooling a different number of SGs (from two to 22). These SGs were randomly chosen among all the sampled SGs in the matrix. *F-*statistics were calculated using R scripts developed by us in the R© software version 3.3.2 (R Core Team 2014). Also, in the simulation study, *F-*statistics were calculated either by distinguishing the age/reproductive status of individuals or by sampling at random as in the empirical study (where the status of individuals was not always identifiable).

The geographic distance among neighbouring SGs (within sites in the real dataset) was calculated by the euclidean distance based on GPS coordinates using the Landscape Genetics Toolbox in ArcGis (Etherington 2011).

## Results

### Deviations from Hardy–Weinberg equilibrium within SGs

**Fig. 3:**
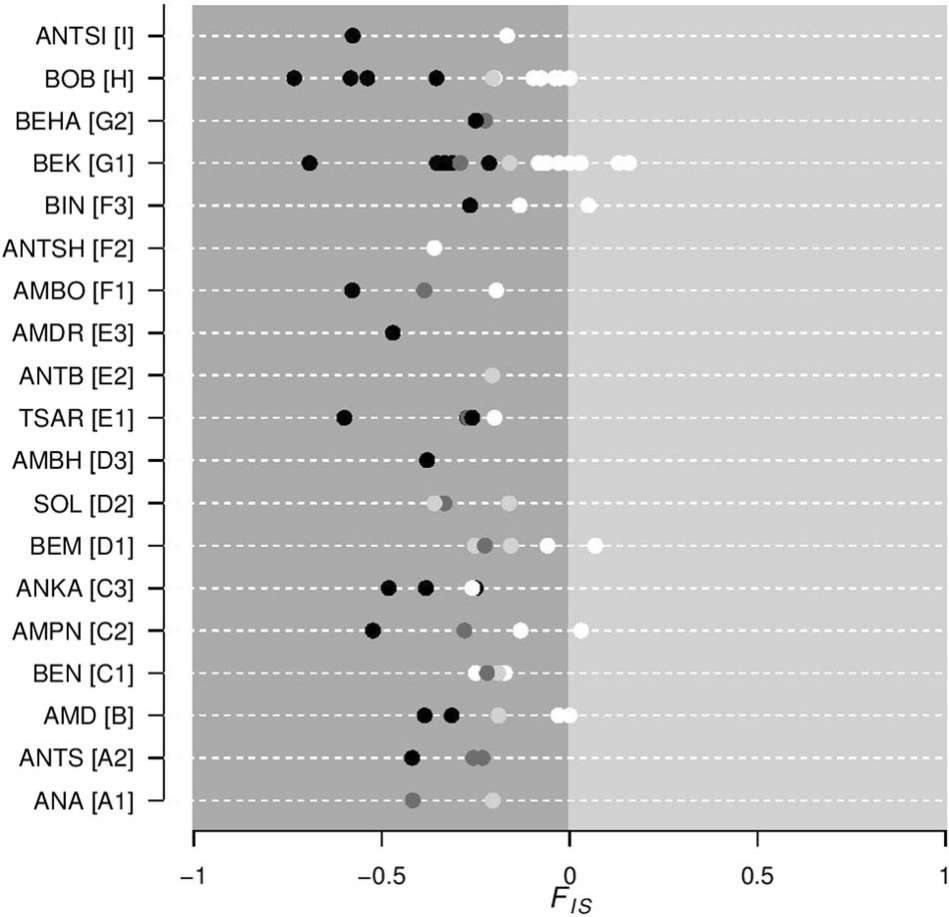
Inbreeding coefficients (*F*_IS_) observed within SGs. Circles are *F*_IS_ values measured within SGs; each dashed horizontal line corresponds to a sampling site (see Fig. 1 for a correspondence between names and geographic locations). Colours correspond to *p*-value significance: white—non-significant, light grey—*p* < 0.05, dark grey—*p* < 0.01 and black—*p* < 0.001. *F*_IS_ were calculated according to Weir and Cockerham (1984) and only SGs where more than one individual was sampled were considered (thus *N* = 76, instead of 103).

**Fig. 4:**
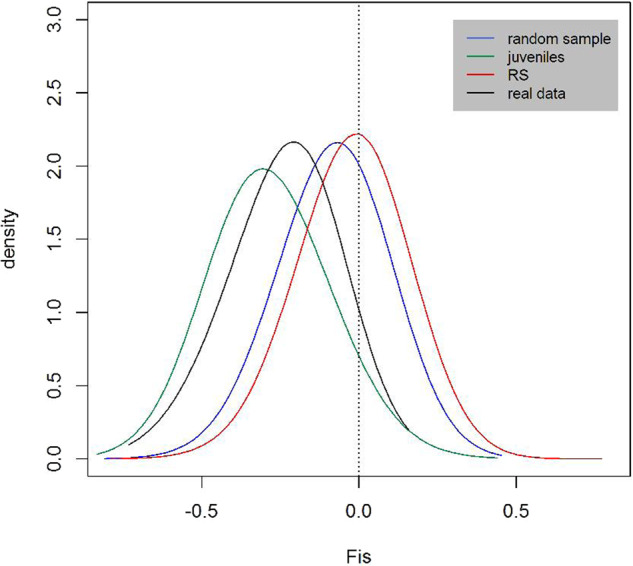
Inbreeding coefficients (*F*_IS_) in real and simulated data. Coloured lines show *F*_IS_ distributions measured in the simulated datasets according to Wright (1951). Different colours represent different sampling schemes—juveniles in green (mean = −0.288; 95% CI: [−0.34, −0.16]), RS in red (mean = −0.016; 95% CI: [−0.06, 0.06]) and random sample in blue (mean = −0.079; 95% CI: [−0.12, 0.01]). Each density curve was obtained using *F*_IS_ values measured at the last time step and from nSG ≥ 2 at 100 independent simulations. This corresponds to 2197 data points when RS individuals are sampled, 1755 data points when only juveniles are sampled, and 2200 points when a random sample was taken. The black curve shows the distribution of *F*_IS_ values obtained from the golden-crowned sifaka real dataset (mean = −0.235; note that these are the same as values shown in Fig. 3). Density curves were fitted using the *locfit* function in the R software. This function estimates the density of a set of values (*F*_IS_) having as limits the minimum and maximum frequencies observed in the dataset and for this reason the density curves shown do not necessarily extent towards zero in the *y* axis.

We found highly negative *F*_IS_ values within SGs both in the empirical and simulation studies, indicating an excess of heterozygotes in relation to what was expected under random mating. In the empirical study, the mean *F*_IS_ was −0.235 (SE = 0.022, range = −0.733 to 0.158) with 86% of SGs (*N* = 65 out of 76) showing negative *F*_IS_ values, out of which 71% (*N* = 46) showed significantly negative *F*_IS_ values (*p* < 0.05). Only 14% (*N* = 11 out of 76) showed null or non-significant positive *F*_IS_ values (Fig. 3). A strong excess of heterozygotes was also found within SGs in the simulation study (Fig. 4). The mean simulated *F*_IS_ per SG was −0.079 (SE = 0.004; range = –0.810 to 0.455) when individuals were sampled at random within SGs (i.e., ignoring their age / reproductive status). This mean *F*_IS_ was measured across 100 repetitions within sampled SGs using SGs where *n* > 1 [${\rm{sample}}\;{\rm{size}} = {\rm{SG}}\left( {n \,>\, 1} \right) \times {{nb.} \rm{repetitions}} = 2200$]. The age/reproductive status of the sampled individuals greatly influenced *F*_IS_ measures: *F*_IS_ were highly negative when sampling only offspring (mean ± SE = −0.288 ± 0.005, range = −0.833 to 0.44; *n* = 1755) indicating a great excess of heterozygotes in the progeny, whereas it was close to zero when only reproductive adults where sampled (mean ± SE = −0.016 ± 0.004, range = −0.793 to 0.773; *n* = 2197; Fig. 4) indicating only a small deficit of heterozygotes in parents. Within SGs, the increase in *F*_IS_ in parents (RS) in comparison to *F*_IS_ in offspring was due to differences in the expected (*H*_E_) rather than observed heterozygosity (*H*_O_), that is *H*_O_ in RS and offspring was quite similar, while *H*_E_ in parents was higher than in offspring (Fig. S1). Altogether, simulations were thus able to recover *F*_IS_ values obtained in the empirical study. We also found that the empirical distribution of *F*_IS_ values was intermediate between the juveniles and the random sample distributions obtained from the simulated dataset (Fig. 4, see also Fig. S2).

### Excess of heterozygotes at different spatial scales

**Fig. 5:**
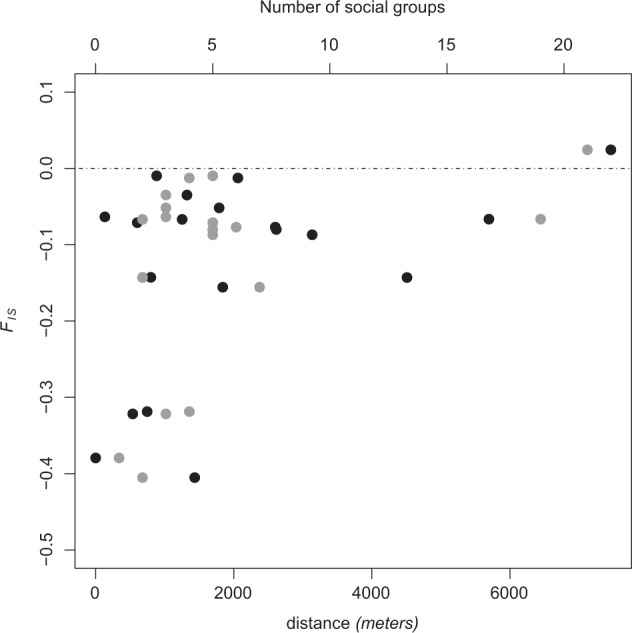
Inbreeding coefficient (*F*_IS_) as a function of the geographic distance and number of SGs pooled. *F*_IS_ measured within sampling sites (Fig. 1). *F*_IS_ within sites (*N* = 19) were measured by pooling neighbouring SGs that belong to a given forest fragment. Black symbols show *F*_IS_ as a function of the number of SGs, whereas grey symbols show *F*_IS_ as a function of the mean geographic distance among the pooled SGs (measured in meters). *F*_ST_ is largely determined by geographically limited dispersal resulting in isolation-by-distance (Wright 1943). Because male sifakas predominantly disperse to neighbouring groups, the observed increase in *F*_IS_ with mean geographic distance was expected (see also Fig. S3).

Even though sifaka SGs taken individually showed a strong excess of heterozygotes (*F*_IS_ < 0), the magnitude of this markedly decreased at the “site” and fragment scales (significant *F*_IS_ at eight out of 19 sites and significant *F*_IS_ at four out of nine fragments, Tables 1 and S2). In other words, *F*_IS_ values tended to become less negative as the hierarchical scale at which samples were analysed increased: mean *F*_IS_ values increased from −0.236 (SGs) to −0.069 (sites, *n* = 19) and −0.044 (fragments, *n* = 9; see Table 1 legend). We found a positive correlation between the increase in *F*_IS_ and the distance between SGs (Figs. 5 and S3): the increase in *F*_IS_ was larger when pooled SGs were located further away from each other, as expected when there is isolation-by-distance (Wright 1943). However, at the scale of the fragment, most SGs are located close to each other and we could not detect any significant correlation between geographic distance and *F*_ST_. Also, our results showed that the magnitude of increase in *F*_IS_ values with changing hierarchical scales depends on the number of SGs pooled at sites and fragments—the larger the number of groups pooled, the greater the increase in *F*_IS_ values (Fig. 5). This was also observed in the simulated dataset, where pooling an increasing number of SGs increased *F*_IS_ towards positive values. In this case, mean *F*_IS_ values were slightly positive when six or ten SGs were pooled and markedly positive only when 22 SGs were pooled together (Fig. S4). This is in agreement with the real dataset results where the largest increase (from −0.137 to 0.025) was found in the BEK site (G1, *N* = 21 pooled SGs). This increase in *F*_IS_ values (SGs to sites and sites to fragments) is consistent with the Wahlund effect and mostly determined by an increase in the expected heterozygosity (both in RS and offspring), rather than by a change in the observed heterozygosity, which was more or less constant among the different hierarchical levels (Table S2 and Fig. S5).

**Table 1:** *F*_IS_ across hierarchical scales (social groups, sites and forest fragments).

Forest fragment	Site	*n SG*	*F*_IS_ *SG*	*F*_IS_ *site*	*F*_IS_ *frg*
ANTSAHARAINGY[A]	ANA[A1]	2 (3)	−0.3***	−0.32***	−0.19***
	ANTS[A2]	3 (7)	−0.27***	−0.16***	
AMPONDRABE[B]^a^	AMD[B]	5 (5)	−0.14***	−0.08*	−0.08*
AMPOETANY[C]	BEN[C1]	5 (6)	−0.24***	−0.08	−0.05**
	AMPN[C2]	4 (5)	−0.16*	−0.01	
	ANKA[C3]	4 (4)	−0.36***	−0.32***	
BEMOKOTY[D]	BEM[D1]	5 (5)	−0.14**	−0.07	−0.03
	SOL[D2]	3 (4)	−0.28***	−0.01	
	AMBH[D3]	1 (1)	−0.38***	−0.38*	
TSARAHITSAKA[E]	TSAR [E1]	5 (5)	−0.31***	−0.09	−0.04
	ANTB [E2]	1 (2)	−0.2*	−0.07	
	AMDR[E3]	1 (2)	−0.51***	−0.41***	
BAA[F]	AMBO[F1]	3 (3)	−0.33***	−0.05	0
	ANTSH[F2]	1 (2)	−0.4	−0.14	
	BIN[F3]	3 (3)	−0.14*	−0.04	
BEKARAOKA[G]	BEK[G1]	16 (21)	−0.1***	0.03	0.01
	BEHA[G2]	2 (2)	−0.26***	−0.14*	
BOBANKORA[H]^a^	BOB[H]	11 (19)	−0.24***	−0.07*	−0.07*
ANTSIASIA[I]^a^	ANTSI[I]	2 (3)	−0.4***	−0.06	−0.06

The first two columns are the names of forest fragments and sites, respectively (see Fig. 1 for a correspondence between names and geographic localities). The third column shows the number of SGs where *n* ≥ 2 (total number of SGs sampled within brackets). *F*_IS_ within SGs was calculated according to Weir and Cockerham (1984). *F*_IS_ at the site and fragment levels were calculated according to Wright (1951). *F*_IS_, the inbreeding coefficient, varies between −1 (all heterozygous) and 1 (all homozygous). This table shows that *F*_IS_ within SGs are negative and values gradually increase towards zero when measured within sites and fragments. This is more easily seen when *F*_IS_ is calculated according to Weir and Cockerham (1984) overall all SGs, sites and fragments: *F*_IS_ (SGs) = −0.236, CI (−0.23 to −0.18); *F*_IS_ (19 sites) = −0.07, CI (−0.09 to −0.05) and *F*_IS_ (fragments) = −0.044, CI (−0.07 to −0.02).

*p < 0.05; **p < 0.01; ***p < 0.001.

^a^Site is the same as fragment—only one value was computed.

## Discussion

The genetic diversity of a population is influenced by its spatial distribution and also by several aspects of population dynamics and behavioural ecology, such as dispersal and mating tactics (Chesser 1991a, b; Sugg et al. 1996; Di Fiore 2012; Parreira and Chikhi 2015). When populations are subdivided in SGs, many individuals are related through parents or through the philopatric sex (thus sharing alleles identical-by-descent through the pedigree). SGs are thus often seen as potentially inbred units that need to evolve active or “passive” mechanisms to counteract potential inbreeding depression effects, such as kin recognition and sex-biased dispersal. However, the subdivision of populations in SGs, while maintaining identity-by-descent sharing through the pedigree, can result in outbreeding (excess in heterozygotes; Chesser 1991a, b; Chesser et al. 1993; Sugg and Chesser 1994; Sugg et al. 1996). Here we show that strongly negative *F*_IS_ values can be the result of social structure driven by age structure (offspring) but that it does not require inbreeding avoidance strategies. Although dispersal is often seen as an inbreeding avoidance mechanism, the fact is that the number of theoretical studies on the relationship between inbreeding and dispersal (Gandon 1999; Perrin and Mazalov 1999; Lehmann and Perrin 2003; Roze and Rousset 2005; Guillaume and Perrin 2006) contrasts with the few empirical studies where dispersal as a means of inbreeding avoidance has actually been quantified (Greenwood et al. 1978; Schiegg et al. 2006; Szulkin and Sheldon 2008). When populations are structured, they are subdivided into distinct units (demes or SGs) among which individuals can move. Thus, dispersal is by definition part of population structure. The fact that individuals move away from their natal unit (deme or SG) and mate elsewhere decreases the chances of mating with close kin thereby decreasing inbreeding.

Negative *F*_IS_ have been reported in various social species, such as in Indian fruit bats (*Cynopterus sphinx*, Storz et al. 2001), yellow*-*bellied marmots (*Marmota flaviventris*, Schwartz and Armitage 1980) and alpine marmots (*Marmota marmota*, Goossens et al. 2001), black-tailed prairie dogs (*Cynomys ludovicianus*, Winterrowd et al. 2009), and white sifakas (*P. verreauxi verreauxi*, Lawler et al. 2003). The consistent excess of heterozygotes found within SGs of the golden-crowned sifaka is considerably higher than comparable estimates for other mammals, suggesting that this species exhibits uncommonly large levels of outbreeding. For instance, the lowest reported *F*_IS_ values were −0.16 in Indian fruit bats and −0.20 in white sifakas, whereas in the present study the most extreme *F*_IS_ value was −0.733 (Fig. 3). We have recently shown in a theoretical study that the negative *F*_IS_ expectation is maximised under a monogamous mating system (Parreira and Chikhi 2015), as seen in golden-crowned sifakas (Meyers 1993). The natural organisation of this species in SGs and the monogamic matings may explain the highly negative values found in the present golden-crowed sifaka study.

### Possible evolutionary consequences of the subdivision into SGs

The simulated datasets revealed differences between *F*_IS_ measured in parents (RS) and offspring meaning that in practice, *inbreeding* is highly dependent on the sampling scheme (Figs. 4 and S2). The difference between inbreeding levels measured in RS and offspring individuals is probably because most RS pairs are composed of individuals that differ in origin and thus carry different alleles. As a consequence, sampling RS individuals increases *F*_IS_ towards zero within SGs (due to the higher *H*_*E*_ in RS; see Fig. S1).

The effect of age and reproductive status can also be interpreted in respect to dispersal timing. Basset et al. (2001) have noted that *F*_IS_ measured in offspring corresponds to sampling individuals before dispersal and thus measures the genetic product of mating (distribution of genes in the next generation), whereas measuring *F*_IS_ in RS individuals corresponds to sampling individuals after dispersal and measures the redistribution of genotypes across the population. For some authors, such as Storz et al. (2001), SGs are transient units from which individuals eventually disperse at some point in their lives. As a consequence, genetic estimates measured at the SG level are quite ephemeral snapshots of the genetic and genotypic diversity of a specific life-cycle time point and are not important on an evolutionary scale. Other authors do not reject the idea that mating and dispersal systems may have an evolutionary importance and SGs have been used to explain fast evolutionary rates supposedly found in mammal genera where most species are social (Bush-Wilson theory; Bush et al. 1977; Wilson 1992). However, this latter argument rests on the assumption that SGs can be treated under the Wright–Fisher framework as small demes. Under the classical framework, SGs are characterised by small effective sizes, high genetic drift and increased homozygosity. This is expected to increase the probability of fixation of new mutations, including chromosomal rearrangements, and accelerate rates of evolution. Our results, and those obtained for other social species exhibiting high levels of individual heterozygosity (consistent with lower drift; Schwartz and Armitage 1980; Pope 1992; Coltman et al. 2003; Lawler et al. 2003), contradict the Bush–Wilson theory. In addition, several recent studies have noted that social structure may influence rates of molecular evolution not because SGs behave as demes but because mating systems may influence the number of DNA replications per generation (Bromham 2009, 2011).

We argue that SGs have a significant effect on instantaneous patterns of genotypic diversity and also that they may have an evolutionary significance different from that predicted by the Bush–Wilson theory. For instance, social structure may have important evolutionary consequences by decreasing the frequency of homozygotes (and maintaining a high genotypic diversity, *H*_*O*_), thus influencing the fitness and survival of individuals and populations.

### Differences in *F*-statistics across geographic scales

We found an excess of heterozygotes in *P. tattersalli* not only within SGs but also in most sites and fragments (Table 1). Although the estimated *F*_IS_ was still negative at several levels of subdivision above the SG, the magnitude of excess of heterozygotes decreased when pooling different SGs (i.e., *F*_IS_ SG < *F*_IS_ site < *F*_IS_ fragment). Importantly, this increase in *F*_IS_ is due to the increase in *H*_*E*_ mainly determined by offspring but also by the classical effect of population structure (Wahlund effect). The Wahlund effect consists in an increase in *F*_IS_ due to the pooling of distinct units of population subdivision (Wahlund 1928) resulting in a heterozygote deficit (*F*_IS_ > 0) when units of population subdivision are panmitic (*F*_IS_ = 0). However, when these units are not at Hardy–Weinberg equilibrium, as observed in SGs of sifakas (*F*_IS_ < 0), the “Wahlund-like” effect may not necessarily result in positive *F*_IS_ and will rather increase *F*_IS_ towards zero. In the golden-crowned sifaka, *F*_IS_ values were highly negative within most SGs (Fig. 3 and Table 1) and that explains why these were still negative at most sites and fragments. Also, the magnitude of the Wahlund effect depends on the number of SGs pooled (Figs. 5 and S4; Table S2) and on the degree of population differentiation. Because (1 − *F*_IT_) = (1 − *F*_ST_)(1 − *F*_IS_), *F*_ST_ values constrain the possible *F*_IS_ and *F*_IT_ values (Wright 1951). SGs are highly complex systems, structured both demographically (composed of individuals with different ages and sexes) and spatially (subdivision into subunits and their spatial arrangement). Observed genetic diversity appears to be influenced by factors intrinsic to the life histories of the organism, such as the mating system and the age structure, and by demographic factors, such as distribution ranges and levels of gene flow. For instance, the increase in *F*_IS_ at the site and fragment scales is determined by the increase in *H*_*E*_ caused by offspring but also depends on a Wahlund effect whose magnitude is largely determined by population structure and levels of subdivision. The several layers of structure interact among each other and quantifying the effect of each level is not easy and calls for more theoretical studies.

## Concluding remarks

In the last decades, there has been an increasing number of studies addressing the fine-scale genetic structure of populations (Lawler et al. 2003; Di Fiore and Fleisher 2005; Fredsted et al. 2005; Archie et al. 2008; Di Fiore 2012; Di Fiore and Valencia 2014; van Djik et al. 2015). Still, compared to the number of studies focusing on the species or “population” scale, there are considerably less studies focusing on the social group. At the “population” and sub-population scales, deviations from random mating are usually not detected. That may be because in population level studies, samples usually consist of an agglomeration of individuals from different SGs and that leads, as we have shown, to an increase in *F*_IS_ towards zero (random mating).

For many population genetics questions, the fact that individuals do not mate at random at finer levels of population structure does not seem problematic if one can still assume that the overall population is, or behaves as if it were, panmitic. This may be true for questions which depend on coalescences (shared common ancestry) occurring in the distant past. In other words, for questions at the level of the population, it may still be possible to use classical coalescent theoretical treatments to infer key evolutionary parameters, such as effective population sizes (*N*_e_), despite the fact that species do not mate at random. However, for questions depending on shared ancestry at the very recent past, such as dispersal or inbreeding, deviations from random mating caused by social structure may be important. Deviations from random mating may also be important when conducting analyses that rely on Hardy–Weinberg equilibrium assumptions, such as STRUCTURE or genotype calling (Peterman et al. 2016; Waples and Anderson 2017).

The consequences of social structure can also be studied through the effect of sampling siblings and family clusters for estimates of genetic diversity, such as effective population sizes, allele frequencies and population differentiation (Peterman et al. 2016, Waples and Anderson 2017). For instance, Waples and Anderson (2017) have shown that, under a non-structured population model, *N*_*e*_ can be biased upwardly when siblings are removed and one single representative per family is kept in the sample but downwardly when some families are over-represented. These authors have shown that unbiased estimates of *N*_*e*_ can eventually be obtained if an “optimal” proportion of siblings is removed; however, this “optimal” number varies according to the mating system. These results may be related to the fact that when sampling siblings, we mostly recover coalescences occurring in the very recent past (with shared ancestry depending also on the mating system), whereas when sampling one individual per family (social group), one mostly recovers coalescences occurring at the more distant past.

Finally, our findings are particularly relevant in the context of Madagascar where habitats are increasingly fragmented and many species have been losing genetic diversity (Craul et al. 2009; Bailey et al. 2016; Hawkins et al. 2018; Vieilledent et al. 2018). The effect of ecological disturbance on social structure in Malagasy species is not well studied. However, studies in Australian social mammals suggest that habitat disruption may affect the structure and function of SGs (Banks et al. 2007). For instance, habitat loss and fragmentation may alter group size and mate availability causing changes in mating and dispersal behaviours. Unfortunately, it may be difficult to fully elucidate certain features of social systems, such as dispersal patterns, kinship structure, and mating system from observational data. In this context, simulation tools, as the one used in the present study, can be applied to other social species of interest. One could for instance, simulate alternative scenarios (using different ecological and life-history parameters and SGs network topology) to identify some of the most important factors affecting genetic and genotypic diversity.

## Supplementary information

Supplementary Material

## Data Availability

The complete genotype microsatellite dataset and the geographic coordinates of all individuals are available in the Dryad Digital Repository: 10.5061/dryad.8f45n. We have excluded six individuals from the original dataset deposited in the Dryad as for these samples analysis could not be performed at the site and fragment scales (ANALABE and AMBORONARIVO). Results for the computer simulations and R-scripts for the analysis can be provided upon request.
