# Supplementary material for: Genetic consequences of social structure in the golden-crowned sifaka
Source: Heredity (Edinb). 2020 Aug 13;125(5):328–39. doi: 10.1038/s41437-020-0345-5 (PMC7555495; doi:10.1038/s41437-020-0345-5)
Supplement: Supplementary file 1 — Supplementary Material [file 41437_2020_345_MOESM1_ESM.pdf]

Genetic consequences of social structure in the golden-crowned sifaka
B. Parreira; E. Quéméré; C. Vanpé; I. Carvalho; L. Chikhi

Supplementary Material

The social structure framework

We used a forward time individual based framework (Parreira and Chikhi 2015). Under this framework, a population is modelled as a network of social groups (SGs) among which individuals can disperse. SGs are relatively small age-structured units where diploid dioceous individuals reproduce according to specific mating strategies, such as monogamy, polygyny, etc. (see below). Within SGs, individuals undergo a simplified life-cycle where important events occur, such as *i)* mating *ii)* aging and death *iii)* migration and *iv)* colonisation. Through the life-cycle, individuals go through different age / reproductive classes (new-borns, juveniles, and adults, see below). Each simulation is divided in discrete time intervals, $t; t+1;\ t+2 \ldots$ in which life-cycle events occur exactly by the order previously mentioned. The length of time between t and $t+1$ is denominated a time step. In practice, a time step is an arbitrary time unit defined by the user that should equal the duration of the shortest event in the life-cycle. This is for computational reasons, the time could eventually be divided into smaller intervals but this would be very inefficient as anything would occur during most of the time steps. In the simulations performed here, biological time is divided in six months' time intervals (weaning age) and all life time parameters (Table S1) are scaled accordingly. This means that a simulation cycle (*i)* mating *ii)* aging and death *iii)* migration and *iv)* colonization) is repeated every six months and seasonal events, such as mating, will not occur has they do not repeat over such small intervals, however the status of individuals in terms of death events and RS status will be upgraded every six months.

Reproduction: Only a few adults take part at reproductive events. These are identified as reproductive status (RS) individuals. Reproduction occurs among RS individuals living in the same SG. The number of RS males and females is fixed and equal for every SG. This number defines the mating strategy. Mating pairs are formed among RS individuals under the assumption that males can mate with several females but females mate only with a single male. This is modelled by a multinomial

distribution where N_f (RS females) are categorized into N_m (males) categories $Mult\left(Nf,\frac{1}{Nm}\right)$. Mating pairs are formed at random, without any behaviour including those intended to minimise mating among related individuals. For instance, an RS male can potentially mate his own daughter if she reaches sexual maturity while he is still RS (see below). Note that this *Mult* distribution does not apply in the case of monogamy, as simulated for the golden crowned-sifaka, as in this specific case there is only one single mating pair per SG. The number of offspring produced by each mating pair follows a Poisson distribution; the average number of offspring *per female* (λ) is decided by the user and the Poisson distribution is truncated at λ+1. Individuals are identified by their sex, reproductive status, age and genotype and are simulated as sequences of unlinked loci. Each individual inherits one allele chosen at random from each parent.

RS individuals maintain their reproductive status until death. This means that a large part of adults will not have the opportunity to mate and will stay as non-RS for their entire lifetime. Although turnover of mating positions is frequent in many primate species, one can interpret mean lifetime as corresponding to reproductive tenure of real individuals.

Death / Aging: In the model, individuals may live for several months or years depending on the species of interest. We distinguish two mortality rates. These act independently at the offspring and adult stages (Figure 2). Offspring death rate is defined by the user as an explicit rate and corresponds to the proportion of individuals that die before weaning. Adult death rate is not defined as an explicit rate and is a consequence of individual's lifetime which follows a truncated Poisson distribution with mean and maximum values decided by the user.

Dispersal: Individuals move to other SGs in order to replace death RS individuals and become RS in the SG they have moved into. That is, dispersal is an actual dispersal event rather than modeled as an explicit rate. Death events release breeding vacancies within a SG. These will be filled by non-RS individuals that will disperse and establish themselves as new RS. Dispersing individuals are randomly chosen among "linked" SGs according to the pre-defined connections in the network, thus allowing

for long-distance migration and spatial structure. The SGs network can be different among males and females, thus allowing to incorporate sex-biased migration (in the case of philopatry, new RS individuals are chosen among non-RS adults within the SG of interest).

The fact that individuals move only to become RS in another SG means that many SGs may include non-RS adults, i.e offspring from previous mating seasons that were not yet able to disperse. In nature, sifaka males disperse or are evicted from their natal group once they reach adulthood, and thus adult non-breeding males (non-RS individuals) are not part of a SG. Keeping non-RS males within a SG in the simulations allow us to keep track of the SG of origin of each individual more efficiently. When analysing simulated data, we can consider only RS individuals, non-RS females and offspring, and avoid sampling non-RS males in order to mimic sampling in real SGs.

Colonisation: When all RS individuals from one sex, RS individuals of the remaining sex lose the reproductive status (becoming non-RS). The SG virtually vanishes and a new SG will be established. A new pair of RS individuals will be chosen at random among the non-RS adults at linked SGs. It is important to note that under the specific case of monogamy (when there is a single RS pair), individuals only move between SGs to establish a new SG.

Initialisation of the simulation: At the beginning of the simulation, SGs are composed by RS individuals only. Initial genotypes are sampled from an allelic frequency distribution. This can be obtained from an empirical dataset or from a theoretical distribution. In the simulations performed here, this allelic frequency distribution was obtained from a Wright-Fisher population with θ=20 simulated under the *ms* computer simulation program (Hudson 2002). Because we simulated 484 SGs with two RS individuals each, when the population is initialised it experiences a bottleneck. We thus allowed simulations under the SGs framework to run around 10,000 generations, far above the time at which we expect the genetic equilibrium to be reached ($4N_e\mu$ generations).

We assumed that mutations were selectively neutral and occurred according to the single stepwise mutation model (SMM, Ohta and Kimura 1973). Each individual was identified by 13 selectively

neutral microsatellite loci, similar to that obtained in the empirical study, with a unique mutation rate of 6 x 10^{-4} mutations per generation / per locus. This mutation rate was found to be within reported mutation rates in humans (Sun et al. 2012) and was chosen so that simulated datasets had levels of genetic diversity similar to what was found in the real dataset.

Supplementary figures and tables:

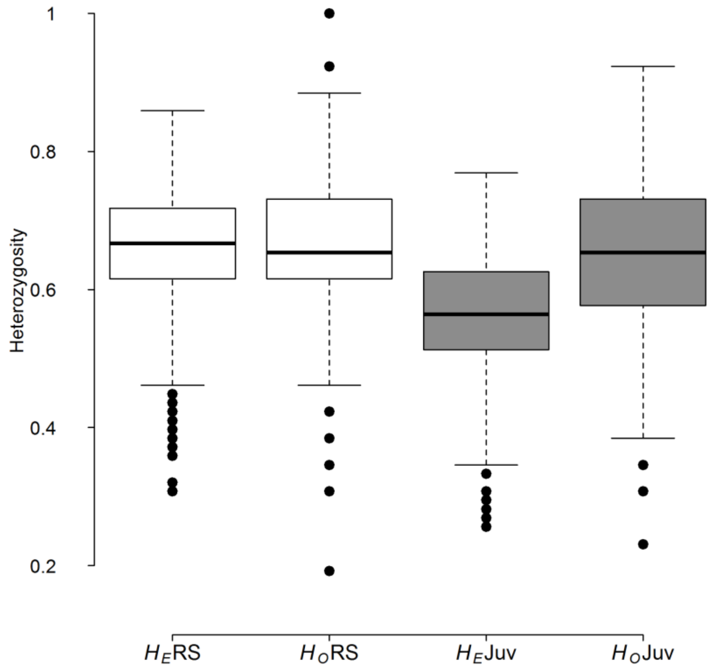

Figure S1. Expected and observed heterozygosity at different age/ reproductive classes.

White boxplots represent expected (H_E) and observed (H_O) heterozygosites measured at RS individuals; grey boxplots represent expected (H_E) and observed (H_O) heterozygosites measured at juveniles (Juv). This plot shows that H_O is similar between classes and that H_E varies, being higher at the RS. The difference between F_{IS} between parents and offspring is thus a consequence of differences in H_E.

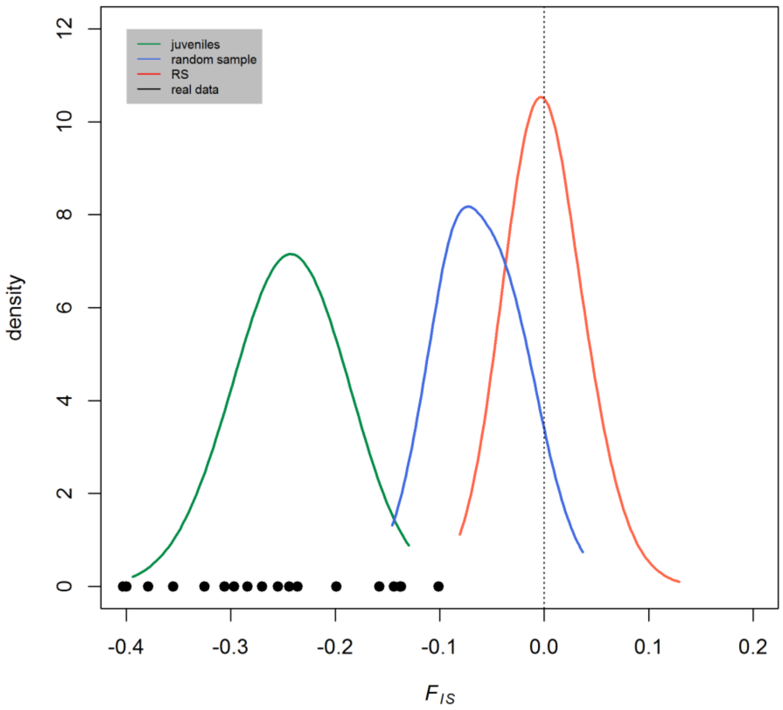

Figure S2. Inbreeding coefficients (F_{IS}) measured in the real and simulated data.

F_{IS} values measured within sampled SGs according to Weir and Cockerham (1984). The black dots are F_{IS} values obtained from the golden-crowned sifaka real dataset. Colored lines are F_{IS} distribution obtained from the simulated datasets; the different colors represent different sampling schemes – juveniles in green (mean=-0.247), RS in red (mean=-0.002) and a random sample in blue (mean=-0.061). The Weir and Cockerham (1984) estimator calculates genetic variance accounting for the unequal sample size obtained at subgroups (SGs) and thus produces an *F-value* that is a weighted mean of within SGs F_{IS}. This figure shows that F_{IS} values obtained in juvenile simulated data fit the empirical F_{IS} values obtained.

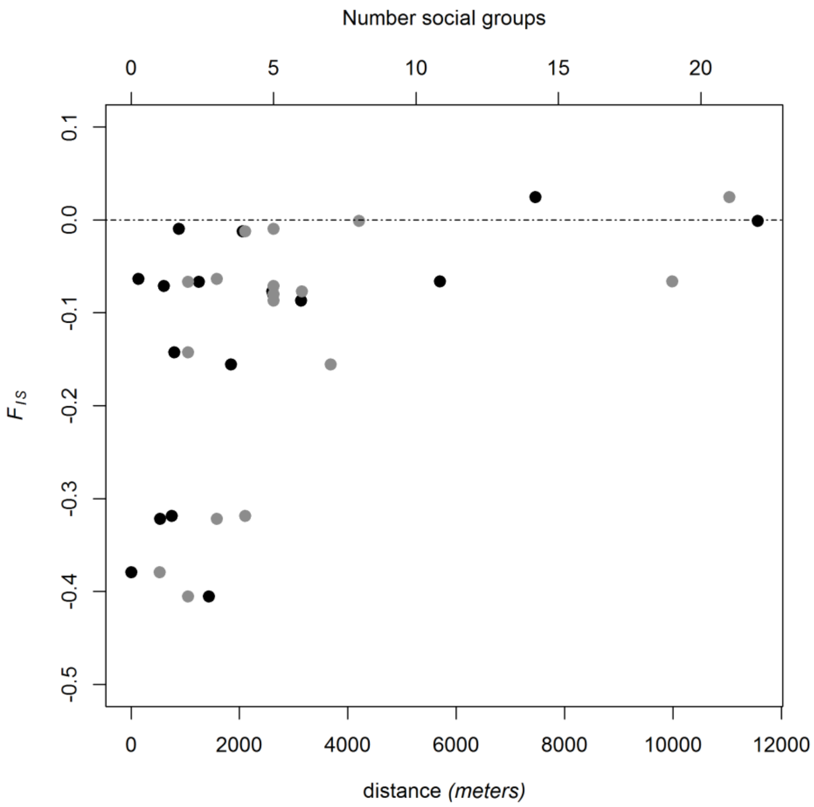

Figure S3. The effect of sampling effort on estimates of inbreeding.

F_{IS} as a function of the geographic distance (measured in meters) and also as a function of the number of SGs within each site. We reanalysed F_{IS} by considering the BAA (F) forest as one single site. This was done because at the time that samples were collected (2006 and 2008), the study area was a more continuous forest than today. This figure shows that when BAA is considered as a single site, the correlation between the number of SGs/ geographic distance and F_{IS} is even more evident.

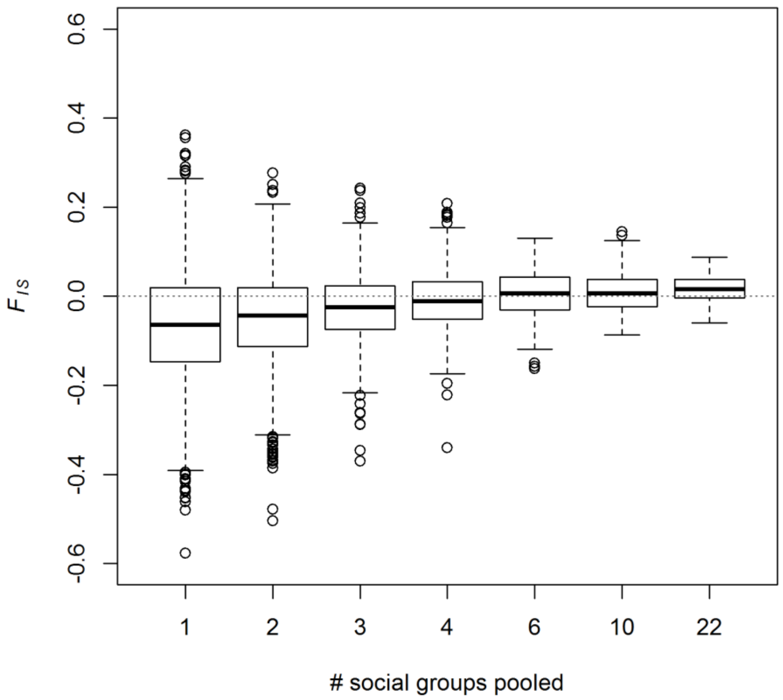

Figure S4. Inbreeding coefficient (F_{IS}) as a function of the number of SGs pooled in the simulation study.

F_{IS} measured in the simulations when a variable number of SGs was pooled. One SG (*x* axis) corresponds to F_{IS} measured within groups (no pooling) whereas all other values (two, three, four, etc.) corresponds to pooling of SGs. These SGs were randomly chosen among all the 484 SGs in the simulated network. F_{IS} values were calculated by sampling at random four individuals among all the age/ reproductive classes within chosen groups. The dotted line refers to the expected F_{IS}(=0) in a panmitic population.

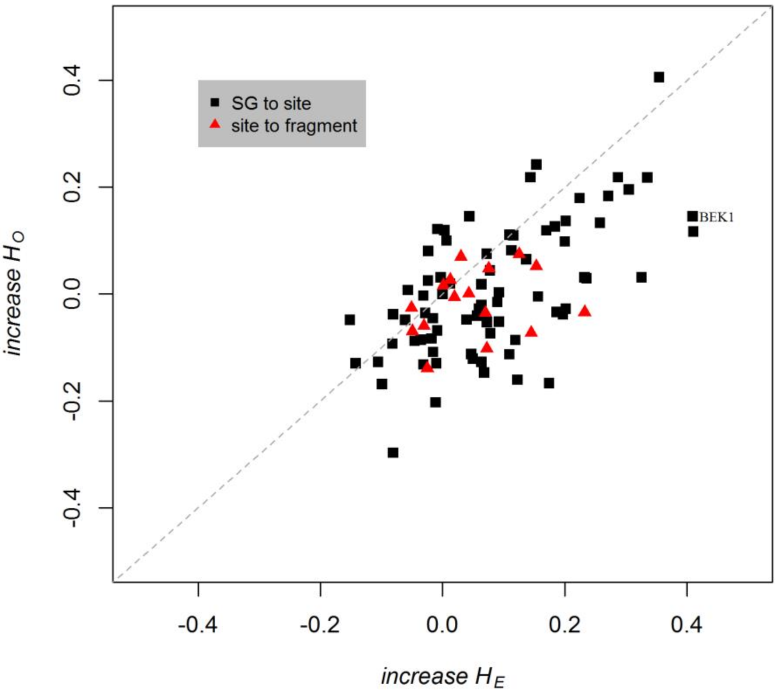

Figure S5. Expected and observed heterozygosity as a function of the sampling unit.

This figures shows the proportion of increase in the expected (H_E) and observed heterozygosity (H_O) from SGs to sites (black dots) and sites to fragments (red dots). Points above zero in the *x* axis show that there is an increase in H_E. Similarly points above zero in the *y* axis show that there is an increase in H_O. In contrary, negative values in both axes show a decrease in heterozygosity from SGs to sites (or sites to fragments). For instance, the point identified shows that there was an increase in 40% on H_E from the SG (BEK1) to the BEKARAOKA fragment (G1, Figure 1), whereas the same increase in H_O was around 15%. The fact that most values are below the dotted line shows that, in general, there is a larger increase in H_E than in H_O from SGs to sites or from sites to fragments. This also shows that the increase in F_{IS} is mostly due to a change in H_E rather than due to changes in H_O.

Table S1. Parameter values as used in the simulations.

Parameter	Values in literature	Range tested	Values used
#females	1	1	1
#males	1	1	1
♀Life span	-	8 – 15 *yr*	12 *yr*
♂life span	-	8 – 15 *yr*	12 *yr*
Maximum life time	20 *yr*	14 – 20 *yr*	15 *yr*
♀first reproductive age	2/ 3 *yr*	4 – 8 *yr*	3 *yr*
♂first reproductive age	2/ 3 *yr*	4 – 8 *yr*	3 *yr*
Weaning age	5-6 *mo*	3–6 *mo*	6 *mo*
Birth interval	2 *yr*	1/ 2 *yr*	2 *yr*
#offspring	1	1 – 2	2
Offspring death rate	-	0.1/0.2	0.1

All results presented throughout this work have been obtained by using the values presented in the third column. The first column shows values reported in the literature, measured in captive or wild populations of golden-crowned sifaka (see also Materials and Methods); the second column shows the range of values that has been tested in order to obtain the final combination of parameters used. *yr* - years old; *mo* – months.

Table S2. *F*-statistics across hierarchical scales (social groups, sites and forest fragments).

	Social Group					*Sites*				*Fragments*			
Site	***n SG***	***H_E (95%CI)***	***H_O (95%CI)***	***F_{IS}***	***F_{ST}***	***H_E (95%CI)***	***H_O (95%CI)***	***F_{IS}***	***F_{ST}***	***H_E (95%CI)***	***H_O (95%CI)***	***F_{IS}***	***F_{ST}***
A1<br>A2	3 (2)<br>7 (3)	0.53 (0.06 – 0.79)<br>0.6 (0.25 – 0.83)	0.66 (0.06-1)<br>0.74 (0.25-1)	-0.3***<br>-0.27***	-0.03<br>0.1**	0.5 (0.15-0.70)<br>0.61 (0.4-0.77)	0.65 (0.15-0.88)<br>0.7 (0.44-0.93)	-0.32***<br>-0.16***	0.03	0.58 (0.36-0.77)	0.69 (0.40-0.91)	-0.18***	0.11***
B†	5 (5)	0.71 (0.5 – 0.86)	0.81 (0.25-1)	-0.14***	0.07*	0.73 (0.61-0.84)	0.79 (0.63-0.94)	-0.08*	–	0.73 (0.61-0.84)	0.79 (0.62-0.94)	-0.08*	
C1<br>C2<br>C3 †	5 (5)<br>5 (4)<br>4 (4)	0.57 (0 – 0.83)<br>0.56 (0 – 0.87<br>0.6 (0 - 0.83	0.67 (0-1)<br>0.63 (0-1)<br>0.73 (0-1)	-0.24***<br>-0.16*<br>-0.35***	0.15***<br>0.15**<br>0.03	0.64 (0.46-0.74)<br>0.63 (00.46-0.79)<br>0.57 (0.34-0.70)	0.68 (0.54-0.84)<br>0.63 (0.38-0.92)<br>0.73 (0.4-1)	-0.08<br>-0.01<br>-0.32***	0.08***	0.65 (0.51-0.73)	0.68 (0.56-0.84)	-0.05**	
D1<br>D2<br>D3	5 (5)<br>4 (3)<br>1 (1)	0.64 (0.33 – 0.93)<br>0.57 (0 – 0.83)<br>0.63 (0.3 – 0.83)	0.69 (0.33 - 1)<br>0.68 (0 - 1)<br>0.77 (0.3 - 1)	-0.14**<br>-0.28***<br>-0.38***	0.07*<br>0.25**	0.65 (0.48-0.80)<br>0.67 (0.57-0.77)<br>0.63 (0.3-0.83)	0.69 (0.46-0.93)<br>0.67 (0.45-0.88)<br>0.77 (0.3-1)	-0.07<br>-0.01<br>-0.38*	0.06**	0.67 (0.53-0.81)	0.69 (0.48-0.85)	-0.03	
E1<br>E2<br>E3	5 (5)<br>2 (1)<br>2(1)	0.54 (0 – 0.83)<br>0.64 (0.51 – 0.84)<br>0.48 (0.2 – 0.73)	0.65 (0 - 1)<br>0.75 (0.5-1)<br>0.64 (0.2-1)	-0.31***<br>-0.2*<br>-0.51***	0.2***<br>0.24<br>0.13	0.62 (0.43-0.81)<br>0.7 (0.54-0.84)<br>0.54 (0.4-0.71)	0.67 (0.4-0.84)<br>0.74 (0.6-0.88)<br>0.71 (0.4-1)	-0.09<br>-0.07<br>-0.41***	0.11***	0.67 (0.5-0.82)	0.7 (0.56-0.83)	-0.05	
F1<br>F2<br>F3	3 (3)<br>2 (1)<br>3 (3)	0.51 (0 - 0. 74)<br>0.54 (0 – 0.83)<br>0.67 (0.33 – 0.83)	0.64 (0-1)<br>0.65 (0-1)<br>0.73 (0 - 1)	-0.33***<br>-0.4<br>-0.14*	0.26***<br>0.25***<br>0.12	0.61 (0.44-0.78)<br>0.65 (0.45-0.83)<br>0.71 (0.58-0.84)	0.64 (0.38-0.93)<br>0.72 (0.33-1)<br>0.74 (0.43-1)	-0.05<br>-0.14<br>-0.03	0.08***	0.69 (0.57-0.83)	0.69 (0.48-0.91)	0	
G1<br>G2	21 (16)<br>2 (2)	0.66 (0 – 0.98)<br>0.71 (0.33 – 1)	0.70 (0-1)<br>0.82 (0.33-1)	-0.1***<br>-0.26***	0.12***<br>0.14***	0.72 (0.57-0.83)<br>0.74 (0.49-0.87)	0.71 (0.52-0.87)<br>0.83 (0.4-1)	0.02<br>-0.14*	*-0.01*	0.72 (0.56-0.82)	0.72 (0.54-0.86)	0.01	
H†	19 (11)	0.62 (0 - 1)	0.72 (0 - 1)	-0.24***	0.15***	0.66 (0.47-0.81)	0.7 (0.51-0.88)	-0.07*	–	0.66 (0.47-0.81)	0.7 (0.51-0.88)	-0.07*	
I†	3 (2)	0.5 (0 – 0.83)	0.63 (0-1)	-0.4***	0.3	0.57 (0.25-0.78)	0.6 (0.27-0.9)	-0.06	–	0.57 (0.25-0.78)	0.6 (00.27-0.90)	-0.06	

The first column represents the denomination of sites. The second column shows the number of SGs sampled – number of SGs where n ≥ 2 and total number of SGs sampled within brackets. H_E and H_O are expected and observed heterozygosity values, respectively and values within brackets refer to 95% confidence interval (these were calculated using the R software). F_{IS}, the inbreeding coefficient, varies between -1 (all heterozygous) and 1 (all homozygous). F_{IS} values presented here are the same as shown in Table 1. This table shows that H_O is higher than H_E at the SG level up to 29%, but this ratio decreases when heterozygosities are measured at the site and fragment scales. This decrease is due to an increase in H_E (SG < site < fragment). F_{ST} values show the differentiation among SGs within a given site and among sites within a given fragment. *$^*p < 0.05$, $^{**}p < 0.01$ and $^{***}p < 0.001$*; † site is the same as fragment – only one value was computed.

References

Hudson RR (2002) Generating Samples under a Wright-Fisher Neutral Model of Genetic Variation. *Bioinformatics* **18**: 337–38.

Ohta T, Kimura M (1973) A model of mutation appropriate to estimate the number of electrophoretically detectable alleles in a finite population. *Genet Res* **89**: 367-370.

Parreira B, Chikhi L (2015) On Some Genetic Consequences of Social Structure, Mating Systems, Dispersal, and Sampling. *PNAS* **112**: E3318–26

Sun JX, Helgason A, Masson G, Ebenesersdóttir SS, Li H, Mallick S, et al. (2012) A direct characterization of human mutation based on microsatellites. *Nat Genet* **44**: 1161–5.

Weir BS, Cockerham CC (1984) Estimating *F*-Statistics for the Analysis of Population Structure. *Evolution* **38**, 1358-1370.
